# Munc13b stimulus-dependently accumulates on granuphilin-mediated, docked granules prior to fusion

**DOI:** 10.1247/csf.22005

**Published:** 2022-04-06

**Authors:** Kouichi Mizuno, Tetsuro Izumi

**Affiliations:** 1 Laboratory of Molecular Endocrinology and Metabolism, Department of Molecular Medicine, Institute for Molecular and Cellular Regulation, Gunma University, Maebashi 371-8512, Japan

**Keywords:** docking, insulin, live cell imaging, priming, TIRF microscopy

## Abstract

The Rab27 effector granuphilin plays an indispensable role in stable docking of secretory granules to the plasma membrane by interacting with the complex of Munc18-1 and the fusion-incompetent, closed form of syntaxins-1~3. Although this process prevents spontaneous granule exocytosis, those docked granules actively fuse in parallel with other undocked granules after stimulation. Therefore, it is postulated that the closed form of syntaxins must be converted into the fusion-competent open form in a stimulus-dependent manner. Although Munc13 family proteins are generally thought to prime docked vesicles by facilitating conformational change in syntaxins, it is unknown which isoform acts in granuphilin-mediated, docked granule exocytosis. In the present study, we show that, although both Munc13a and Munc13b are expressed in mouse pancreatic islets and their beta-cell line MIN6, the silencing of Munc13b, but not that of Munc13a, severely affects glucose-induced insulin secretion. Furthermore, Munc13b accumulates on a subset of granules beneath the plasma membrane just prior to fusion during stimulation, whereas Munc13a is translocated to the plasma membrane where granules do not exist. When fluorescently labeled granuphilin was introduced to discriminate between molecularly docked granules and other undocked granules in living cells, Munc13b downregulation was observed to preferentially decrease the fusion of granuphilin-positive granules immobilized to the plasma membrane. These findings suggest that Munc13b promotes insulin exocytosis by clustering on molecularly docked granules in a stimulus-dependent manner.

## Introduction

The Rab27 effector granuphilin is considered to be essential for the docking of secretory granules to the plasma membrane, because granuphilin-knockout (KO) mouse pancreatic beta cells and pituitary endocrine cells show almost no granules attached to the plasma membrane under electron microscopy (Gomi *et al.*, 2005, 2007). Granuphilin appears to function by linking both Rab27 on the granule membrane and syntaxin as fusion machinery on the plasma membrane (Torii *et al.*, 2004). However, granuphilin-null beta cells paradoxically increase insulin secretion in both resting and evoked states despite the granule docking defect. This is because it specifically interacts with and stabilizes the fusion-incompetent, closed form of syntaxins 1~3 (Fukuda *et al.*, 2005; Torii *et al.*, 2002; Wang *et al.*, 2011), which alone cannot form a complex with other SNARE proteins to mediate fusion reactions. Thus, granuphilin likely serves a gating function by preventing spontaneous fusion in a resting state and unlimited fusion in an evoked state by stalling incoming granules in the docking state (Izumi, 2021). This control is essential for regulated exocytosis, in which bioactive substances are released only in the presence of secretagogues. It should be noted, however, that the docked granules are not inert, and are instead actively fused after stimulation, although the probability of them fusing is lower than that of neighboring granules that are untethered by granuphilin (Mizuno *et al.*, 2016). Therefore, there must be a mechanism that converts the closed form of granuphilin-associated syntaxin to the open form in a stimulus-dependent manner. The Munc13 family proteins are generally involved in this transition step from the closed syntaxin–Munc18 complex to the SNARE assembly in secretory pathways (Ma *et al.*, 2011; Richmond *et al.*, 2001). It has been shown that Munc13a-deficiency eliminates fusion competence in most glutamatergic neurons (Augustin *et al.*, 1999). On the other hand, Munc13a homodeficiency or haplodeficiency only partially decreases glucose-induced insulin secretion (GSIS), which is mainly attributed to the decrease in newly recruited granules in the sustained late phase after stimulation (Kang *et al.*, 2006; Xie *et al.*, 2012). Therefore, it is possible that other members of Munc13 proteins, such as Munc13b–d, are also involved in insulin granule exocytosis and granuphilin-mediated, docked granule exocytosis. Here, we investigate the effect of Munc13 isoform deficiency in living beta cells using total internal reflection fluorescence (TIRF) microscopy, which can distinguish various granule prefusion behaviors. As a result, we show that Munc13b stimulus-dependently accumulates on the granules docked to the plasma membrane by granuphilin and specifically promotes their fusion.

## Materials and Methods

### Immunoprocedures

Immunoblotting, immunoprecipitation, and immunostaining analyses were performed as described previously (Mizuno *et al.*, 2011) using the following primary antibodies. Anti-granuphilin rabbit polyclonal antibody (αGrp-N) was generated as described previously (Yi *et al.*, 2002). Anti-Munc13d rabbit polyclonal antibody (Shirakawa *et al.*, 2004) was provided by Dr. H. Horiuchi (Tohoku University). Anti-panMunc13 and anti-calnexin antibodies were purchased from BD Transduction Laboratories (Franklin Lakes, NJ, USA). Anti-red fluorescent protein (RFP) and anti-green fluorescent protein (GFP) were purchased from MBL International (Woburn, MA, USA). GFP-nanobody (Katoh *et al.*, 2015) and mCherry-nanobody (Katoh *et al.*, 2016) are gifts from Drs. Y. Katoh and K. Nakayama (Kyoto University). Anti-RIM1/2 antibody was purchased from Synaptic Systems (Goettingen, Germany). Quantification of immunoblot signals was performed using ImageQuant TL software (Cytiva, Marlborough, MA, USA). A batch insulin secretion assay was performed as described previously (Mizuno *et al.*, 2011). Insulin was measured using an AlphaLISA insulin kit (PerkinElmer; Waltham, MA, USA) or an Insulin High Range Assay kit (Cisbio; Codolet, France).

### Animal procedures

All animal experiments were performed according to the rules and regulations of the Animal Care and Experimental Committees of Gunma University. C57BL/6 mice were originally purchased from CLEA Japan (Tokyo, Japan). *Jinx* mice with the Munc13d mutation (Crozat *et al.*, 2007) were purchased from the University of California, Davis via Mutant Mouse Regional Resources Centers. Pancreatic islets were isolated as described previously (Wang *et al.*, 2011).

### Cell culture and siRNA treatment

The granuphilin-deficient beta cell lines were previously generated from granuphilin KO mice (Mizuno *et al.*, 2016). MIN6 cells and their derivative cells were cultured in high-glucose (25 mmol/L) Dulbecco’s modified Eagle’s medium supplemented with 15% fetal calf serum and 55 μmol/L 2-mercaptoethanol in a humidified incubator with 95% air and 5% CO_2_ at 37°C. MIN6 cells were transfected with 50 nmol/L On-Target plus SMARTpool siRNAs (Dharmacon; Lafayette, LA, USA) for mouse Munc13a (L-061229-01), Munc13b (L-048463-01), and non-targeting control siRNA pool (D-001810-10) using Lipofectamine RNAiMAX reagent (Thermo Fisher Scientific; MA, Waltham, USA). Cells were given the second transfection 3 days after the first transfection. Three days after the second transfection, they were used for experiments. Non-transfected cells were also used as controls.

### Generation of CRISPR/Cas9-mediated Munc13a and Munc13b KO MIN6 cells

Munc13s KO MIN6 cells were generated using the CRISPR/Cas9 system. Two target guide sequences were designed for each Munc13 isoform using a web-based design tool (http://crispr.dbcls.jp/): CAACACATACGTGACGCTGAAGG and GCTGAATGCCATGCGTGACCAGG for Munc13a and AGCACAACTGTAGCAGTTCGTGG and ACTGAAGACAATCCGTCAGTCGG for Munc13b, with PAM sequences (underlines). The oligo pairs encoding the guide sequences were annealed and ligated into a plasmid, pSpCas9(BB)-2A-Puro (PX459; Addgene #48139). The resulting plasmids were transfected into MIN6 cells using Lipofectamine 2000. After 48 h, selection medium containing puromycin (5 μg/ml, InvivoGen; San Diego, CA, USA) was added to the cells and maintained for a further 3 days. The remaining cells were maintained for an additional 10 days to select the cell colonies. Individual cell colonies were collected and a small portion of cells from each clone was used for the genomic DNA extraction to assess the genome editing validation using T7 Endonuclease I (Nippon Gene; Tokyo, Japan). The disruption of Munc13s gene was verified by genomic DNA sequencing and immunoblotting (Supplementary Fig. 3, Fig. 2A).

### Construction of plasmids and recombinant adenoviruses

The construction of Insulin-Venus and Kusabira Orange-1 (KuO)-Granuphilin was previously described (Mizuno *et al.*, 2016, 2011). Mouse Munc13a and Munc13b cDNAs were derived from MIN6 cells. Munc13b cDNA was subcloned into pmCherry-N1 expression vector (Takara Bio Inc.; Kusatsu, Japan) using SalI and SacII sites. For the generation of Munc13b-Venus cDNA, mCherry cDNA of pMunc13b-mCherry was replaced with Venus cDNA prepared by SacII and NotI digestion of pNPY-Venus-N1 (a gift from Dr. A. Miyawaki, RIKEN Brain Science Institute). Munc13b-Cherry or Munc13b-Venus cDNA was subcloned into pENTR 3C Dual Selection Vector (Thermo Fisher Scientific) using SalI and NotI sites. For generation of Munc13a-Cherry cDNA, Munc13b cDNA of pENTR/Munc13b-mCherry was replaced with Munc13a cDNA by a seamless DNA cloning method using SLiCE (Motohashi, 2015). The adenovirus was made using the ViraPower Adenoviral Gateway Expression Kit (Thermo Fisher Scientific). Recombination between the entry plasmid and pAdCMVV5-DEST Vector (Thermo Fisher Scientific) was catalyzed by LR clonase II. For generation of the recombinant adenovirus, pAd/Munc13b-Cherry, pAd/Munc13b-Venus, or pAd/Munc13a-Cherry was transfected into HEK293 cells.

### Microscopy

Confocal and TIRF microscopy was performed as described previously (Mizuno *et al.*, 2016, 2011). Briefly, exocytosis events were manually picked up based on the flash due to neutralization of the intragranular lumen followed by a decrease due to diffusion of Insulin-Venus fluorescence. Exocytosis was classified into 3 types: resident, visitor, and passenger, based on granule visibility before fusion, as shown in Supplementary Fig. 2. For the fluorescence intensity profile analysis during exocytosis, a 4×4 pixels square (1 pixel equals 0.16 μm) was centered on the fusing granules, and the average fluorescence intensity was measured. The local background fluorescence near the fusing granule was subtracted by setting a cellular region with the same square size. The measurement of insulin granule motion, trajectory, and 2D diffusion coefficient was performed under TIRF microscopy using Volocity software (PerkinElmer), as described previously (Mizuno *et al.*, 2016).

### Statistical analysis

All quantitative data are expressed as means±SEM. The significance of differences was assessed by the indicated methods using GraphPad Prism software.

## Results

### Munc13b plays a fundamental role in exocytosis from granules stably residing beneath the plasma membrane

**Fig. 1:**
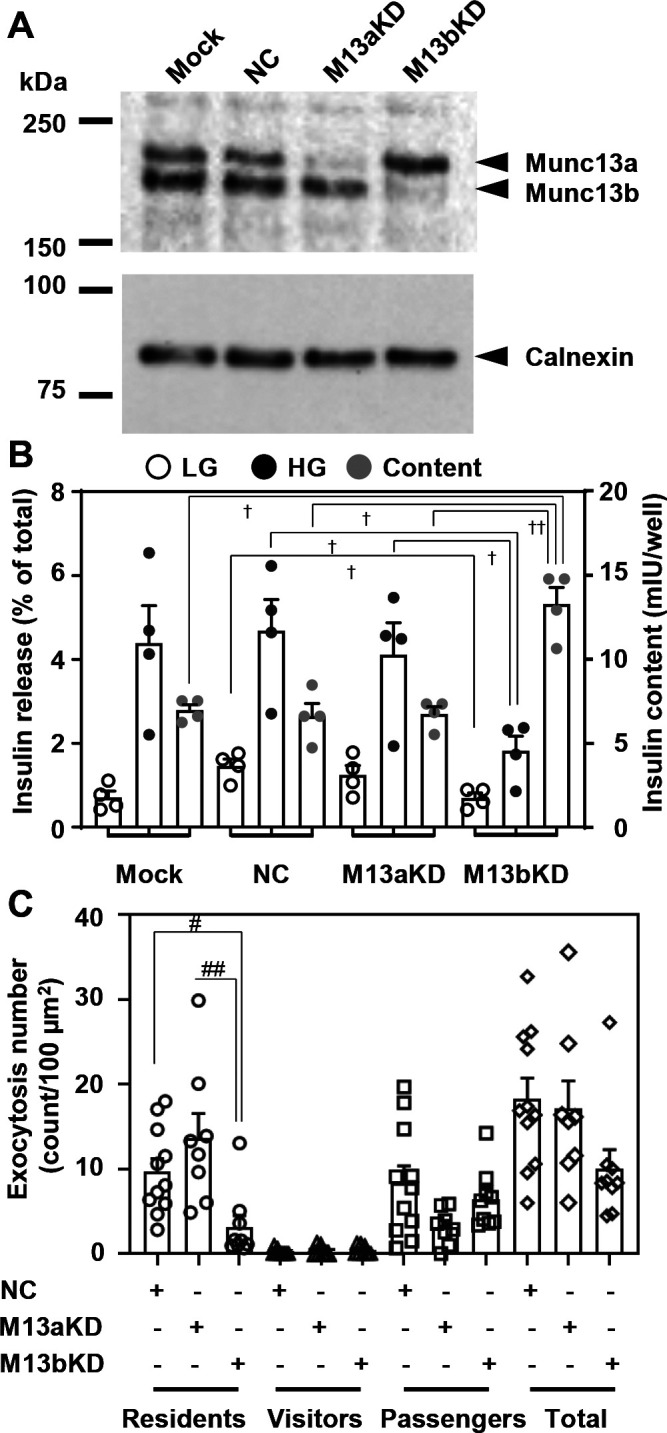
Knockdown (KD) of Munc13b decreases insulin secretion specifically from granules stably residing beneath the plasma membrane A: MIN6 cells were treated without (Mock) or with non-targeting control siRNA (NC) or siRNA against Munc13a (M13aKD) or Munc13b (M13bKD). The protein extracts (50 μg) were electrophoresed for immunoblotting with anti-panMunc13 and anti-calnexin antibodies (left). Note that the upper 210 kDa- and lower 180 kDa-bands are specifically downregulated by treatment of siRNA against Munc13a and Munc13b, respectively. Densitometric analysis of immunoblots revealed that the knockdown efficiencies for Munc13a and Munc13b are 73.8% and 90.4%, respectively (*n*=2). B: MIN6 cells were treated with siRNAs as in A. The cells were preincubated in 2.8 mmol/L low glucose (LG)-containing Krebs-Ringer bicarbonate (KRB) buffer for 30 min and were incubated in LG or 25 mmol/L high glucose (HG) KRB buffer for 20 min. Insulin levels secreted in the media and left in the cell extracts were measured, and their ratios are shown. Insulin levels left in the cell extracts are also shown as insulin content. All experiments were performed in triplicate for each condition and the average values are compared (*n*=4 experiments). ^†^*P*<0.05, ^††^*P*<0.01 by one-way repeated measurement ANOVA followed by post hoc Tukey’s multiple comparisons test. C: MIN6 cells treated with siRNAs were infected with adenovirus expressing Insulin-Venus. Fusion events during HG stimulation for 20 min were manually counted under TIRF microscopy and were categorized into three types as described in Materials and Methods (*n*=8~11 cells from 3 experiments). ^#^*P*<0.05, ^##^*P*<0.01 by one-way ANOVA followed by post hoc Tukey’s multiple comparisons test.

We first examined which isoforms of Munc13 are expressed in mouse pancreatic islets and their beta cell line MIN6 using pan-Munc13 antibody that recognizes Munc13a-c. The two protein bands were detected, although the lower band was predominant in islet cells (Fig. 1A, Supplementary Fig. 1A). The upper 210 kDa- and the lower 180 kDa-bands were taken to correspond to Munc13a and Munc13b, respectively, because each band disappeared or decreased following treatment with the isoform-specific siRNA. Although this antibody does not recognize another isoform Munc13d, the analysis using Munc13d-specific antibody indicated that Munc13d is not significantly expressed in mouse pancreatic islets (Supplementary Fig. 1B). Because the knockdown (KD) was incomplete in islet cells, we thereafter investigated the effects of Munc13a and Munc13b silencing in MIN6 cells. As shown in Fig. 1B, Munc13b KD, but not Munc13a KD, markedly decreased GSIS, suggesting that Munc13b plays a major role in insulin granule exocytosis. We next monitored the fusion behaviors of fluorescently labeled insulin granules in living cells under TIRF microscopy that specifically illuminates fluorophores beneath the plasma membrane. Three distinct types of exocytosis have been identified in pancreatic beta cells (Kasai *et al.*, 2008; Ohara-Imaizumi *et al.*, 2004; Shibasaki *et al.*, 2007): granules visible before stimulation (residents), those that become visible during stimulation (visitors), and those that remain invisible until fusion (passengers; see Supplementary Fig. 2). We found that Munc13b downregulation specifically decreases the resident type exocytosis (Fig. 1C). In contrast, Munc13a downregulation tended to decrease the passenger type exocytosis, consistent with the previous finding (Xie *et al.*, 2012).

**Fig. 2:**
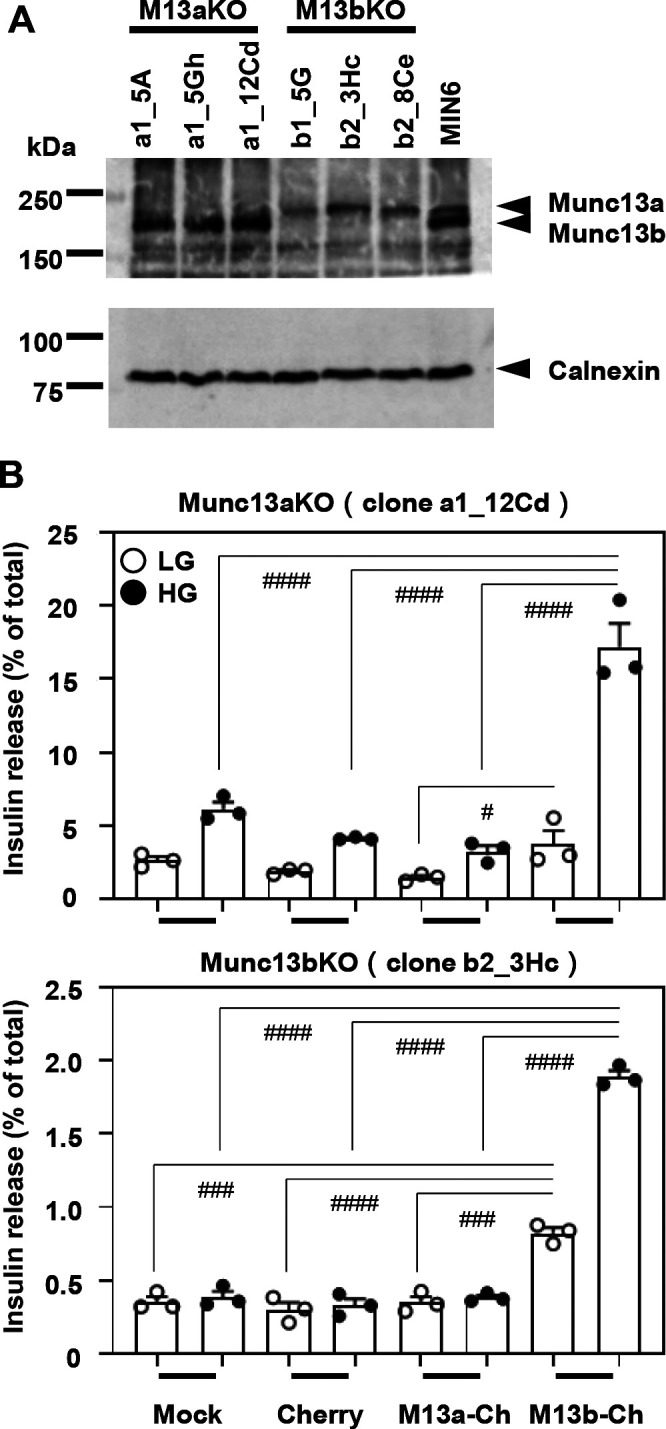
Absence of Munc13b eliminates GSIS A: MIN6 cell lines lacking Munc13a or Munc13b were generated using the CRISPR-Cas9 system. The protein extracts from each of the three lines as well as parental MIN6 cells were electrophoresed for immunoblotting as in Fig. 1A. B: MIN6 cell lines lacking Munc13a (clone a1_12Cd; Munc13aKO cells) or Munc13b (clone b2_3Hc; Munc13bKO cells) were infected by adenoviruses expressing Cherry, Munc13a-Cherry (M13a-Ch), or Munc13b-Cherry (M13b-Ch) under the conditions described in Supplementary Fig. 5. The cells were subjected to insulin secretion assays as described in Fig. 1B (*n*=3). Note that expression of M13b-Ch, but not of M13a-Ch, augments (upper) or recovers (lower) GSIS in Munc13aKO and Munc13bKO cells, respectively. ^#^*P*<0.05, ^###^*P*<0.001, ^####^*P*<0.0001 by one-way ANOVA followed by post hoc Tukey’s multiple comparisons test.

To further investigate the effect of Munc13 isoform deficiency, we generated MIN6 cell lines in which either the Munc13a or Munc13b gene is knocked out using the CRISPR-Cas9 system (Supplementary Fig. 3). This confirmed the isoform-specific loss of protein expression in each cell clone (Fig. 2A). As expected from the previous finding that MIN6 sublines display variable secretory capacities (Minami *et al.*, 2000), each clone released different amounts of insulin. However, all the Munc13b KO cell clones examined here lost the ability to increase insulin secretion in response to glucose stimulation, although the Munc13aKO cell clones preserved it to some extent (Fig. 2B, Supplementary Fig. 4). We then performed rescue experiments to investigate the effect of Munc13 knockin (KI) in these cells. We used MIN6 cells to individually express Munc13a-Cherry or Munc13b-Cherry to the level corresponding to the endogenous level of Munc13b, which was approximately 2~3-fold higher than that of Munc13a (Supplementary Fig. 5). Under this condition, the expression of Munc13b augments GSIS in Munc13aKO cells and restores GSIS in Munc13bKO cells, whereas that of Munc13a has no effect on GSIS in either Munc13aKO or Munc13bKO cells (Fig. 2B, Supplementary Fig. 4). These findings again suggested that Munc13b primarily functions in insulin secretion.

### Munc13b accumulated on granules immobilized on the plasma membrane prior to fusion

**Fig. 3:**
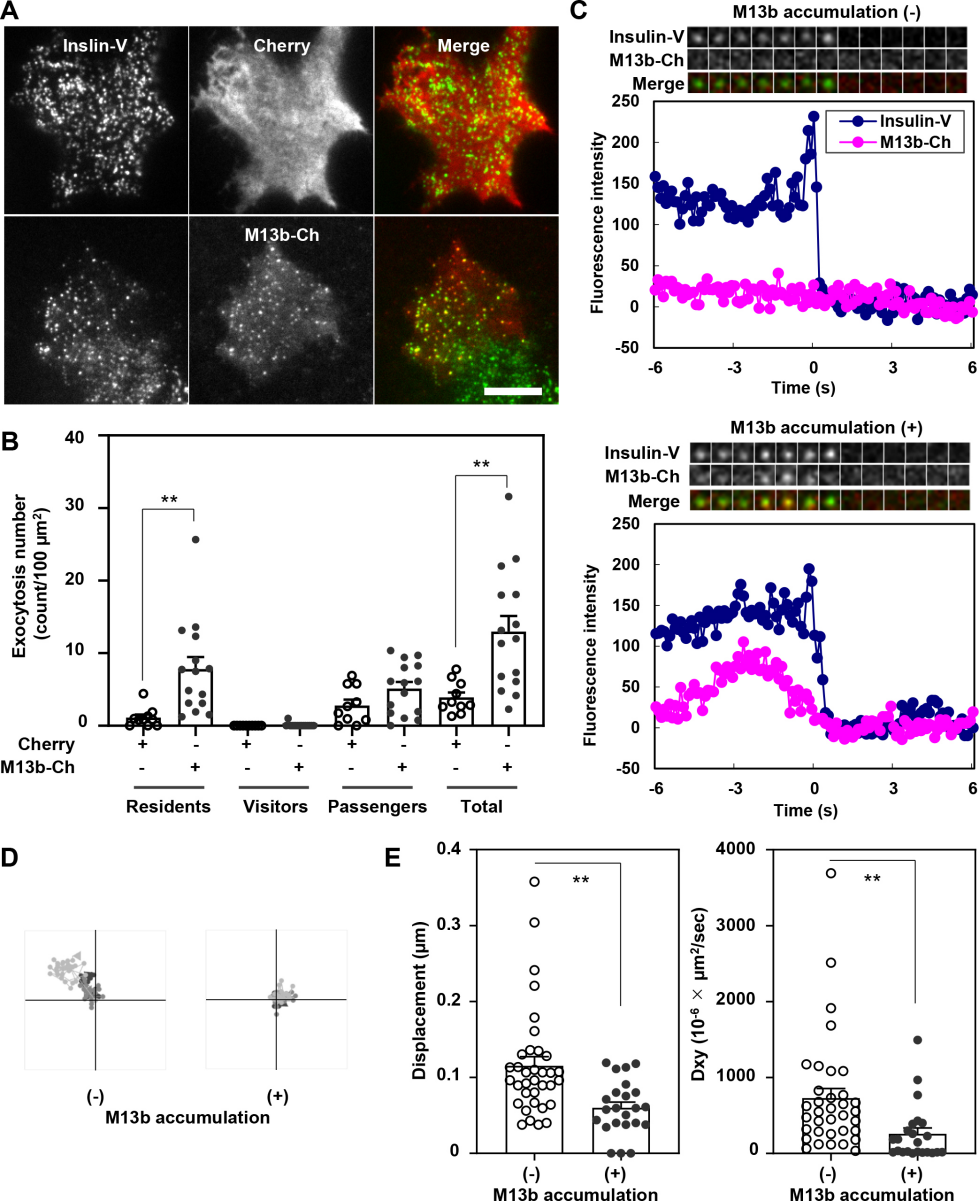
Munc13b accumulates on immobilized granules beneath the plasma membrane prior to fusion A: Munc13bKO cells coexpressing Insulin-Venus (V) and either Cherry or Munc13b-Cherry (M13b-Ch) were observed using TIRF microscopy. Bar, 10 μm. B: Fusion events during 25 mmol/L glucose stimulation for 20 min were counted and categorized as described in Fig. 1C (*n*=10 cells expressing Cherry and *n*=15 cells expressing Munc13b-Cherry from 4 experiments). C: Representative fluorescence intensity profiles of Insulin-V (blue) and M13b-Ch (red) just before and after resident type exocytosis without (upper) or with (lower) M13b-Ch accumulation during 60 mM KCl depolarization stimulation. A kymograph is also shown at the top of each panel. The size of each frame is 6×6 pixels (1 pixel equals 0.16 μm) and the time interval is 1 s. D: Representative 30 time point granule tracks from -5 s to -2 s before fusion without (left) or with (right) M13b-Ch accumulation (area, 1×1 μm^2^; *n*=3 each). E: The displacement and 2D diffusion coefficient Dx,y were calculated from the tracks of fused granules without (*n*=35) or with (*n*=23) M13b-Ch accumulation from 3 experiments. **P<0.01 by unpaired *t* test.

We next examined the intracellular distributions of Munc13a-Cherry and Munc13b-Cherry introduced into Munc13aKO and Munc13bKO cells expressing Insulin-Venus, respectively. Under TIRF microscopy, Munc13a was localized in the cytosol before stimulation, but accumulated onto the plasma membrane, where Insulin-Venus was not colocalized, after stimulation (Supplementary Fig. 6A). In contrast, although Munc13b was also mainly located in the cytosol, it was partly distributed on insulin granules beneath the plasma membrane (Fig. 3A). Furthermore, the expression of Munc13b tended to decrease the number of visible granules, consistent with the view that it promotes their exocytosis. In fact, Munc13b-Cherry KI promoted fusion events primarily attributed to the increase in the resident type exocytosis (Fig. 3B). To understand how Munc13b functions during exocytosis, we observed Munc13b-Cherry together with Insulin-Venus under dual-color TIRF microscopy. To avoid fluorescence quenching during monitoring, we applied KCl depolarization stimulation to the cells for 5 min, instead of glucose stimulation for 20 min, which significantly reduces the time interval between stimulation and fusion. Among the 154 resident type exocytic events we detected, we picked up 58 events that were well separated from other events. We noticed that 40% (23/58 events) accompanied the increase in Munc13b-Cherry fluorescence (1.9-fold increase in the mean intensity at the peak 141.2±14.9 compared with that at the base 73.4±12.8) prior to fusion (2.84±0.35 s before fusion; Fig. 3C, compare Supplementary Videos 1 and 2). In contrast, there was no such accumulation of Munc13a-Cherry on granules showing the resident type exocytosis (Supplementary Fig. 6B). Our previous observations using live cell imaging of insulin granules and the docking machinery granuphilin under TIRF microscopy indicated that the visible granules beneath the plasma membrane can be characterized, in the case of granuphilin-positive granules, as immobile and relatively fusion-reluctant granules, while granuphilin-negative granules are comparatively mobile and fusion-prone (Mizuno *et al.*, 2016). We speculated that the granules accumulating Munc13b correspond to granuphilin-positive, molecularly tethered granules, which requires a conformational change of the associated syntaxins for fusion. Consistent with this expectation, granules with the Munc13b accumulation were immobile compared with those without it (Fig. 3D, E). These findings suggest that Munc13b specifically acts on granules tethered to the plasma membrane by granuphilin.

### Munc13b functions on granuphilin-mediated, docked granules

**Fig. 4:**
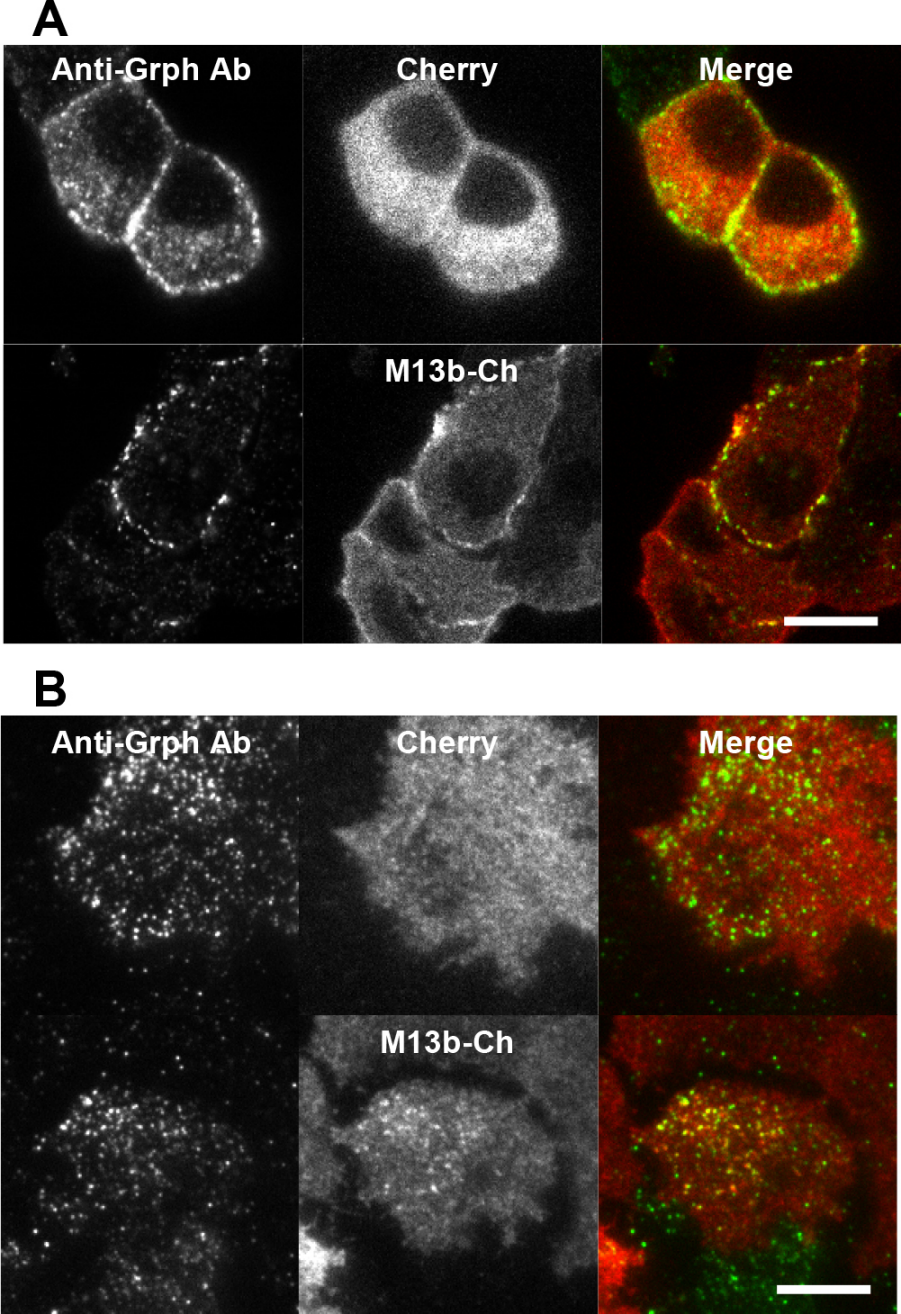
Munc13b is colocalized with granuphilin-positive granules beneath the plasma membrane Munc13bKO cells expressing Cherry or Munc13b-Cherry (M13b-Ch) were immunostained with anti-granuphilin antibody (Grph-Ab), and were observed under confocal (A) or TIRF (B) microscopy. Bars, 10 μm.

**Fig. 5:**
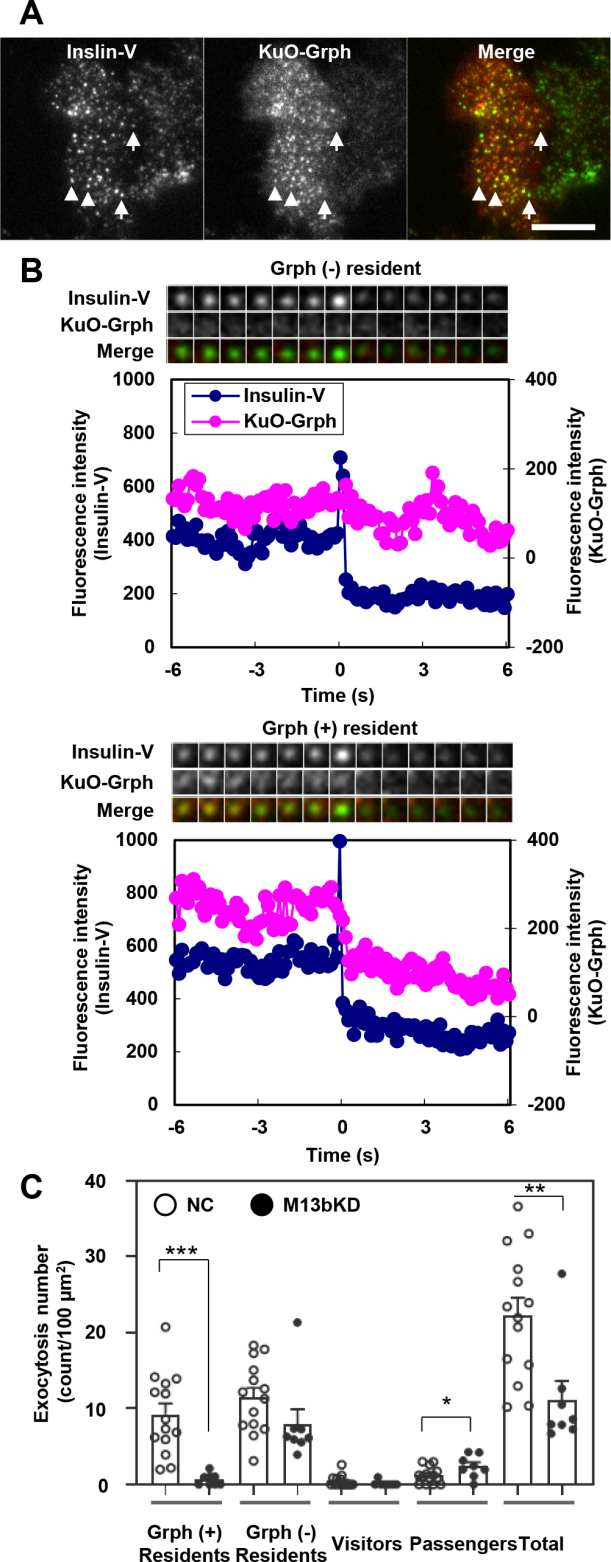
Munc13b deficiency specifically decreases exocytosis of granuphilin-positive granules beneath the plasma membrane A: Granuphilin-null beta cells were infected with adenoviruses expressing Insulin-Venus (V) and KuO-Granuphilin (Grph), and were observed under TIRF microscopy. Note that the majority of visible granules were granuphilin-positive (arrowheads), although some were granuphilin-negative (arrows). Bar, 10 μm. B: Representative fluorescence intensity profiles of Insulin-V (blue) and KuO-Grph (red) just before and after resident type exocytosis from Grph-negative (upper) or -positive (lower) granules during 60 mM KCl depolarization stimulation. A kymograph is also shown at the top of each panel. C: Granuphilin-null cells were treated with non-targeting control siRNA (*n*=15) or siRNA against Munc13b (M13bKD; *n*=10 cells from 5 experiments) as described in Fig. 1C. The cells were then infected with adenoviruses expressing Insulin-V and KuO-Grph as in A. Fusion events during 60 mM KCl stimulation for 5 min were counted and categorized. For the resident type exocytosis, granuphilin-positive and -negative granules were discriminated. *P<0.05, **P<0.005, ***P<0.001 by unpaired *t* test.

Consistent with the above idea, both confocal and TIRF microscopy revealed that a subset of Munc13b-Cherry is clearly colocalized with endogenous granuphilin residing on granules beneath the plasma membrane (Fig. 4A, B). We monitored granuphilin-positive granules in live cells by expressing KuO-Granuphilin. Because overexpression of granuphilin markedly suppresses insulin secretion and massively accumulates granules below the plasma membrane (Torii *et al.*, 2004, 2002), we expressed KuO-Granuphilin at the endogenous level in the beta cell line we previously generated from granuphilin-KO mice (Mizuno *et al.*, 2016). We confirmed that KuO-Granuphilin is associated with the majority of visible granules under TIRF microscopy (Fig. 5A). Because this cell line did not respond well to glucose stimulation, we investigated granule exocytosis in response to high KCl-induced depolarization. As described previously (Mizuno *et al.*, 2016), the fluorescence of KuO-Granuphilin was abruptly decreased from granuphilin-positive granules upon the resident type exocytosis due to lateral diffusion along the plasma membrane (Fig. 5B). Munc13b KD specifically and prominently eliminated the resident type exocytosis from granuphilin-positive granules (Fig. 5C).

**Fig. 6:**
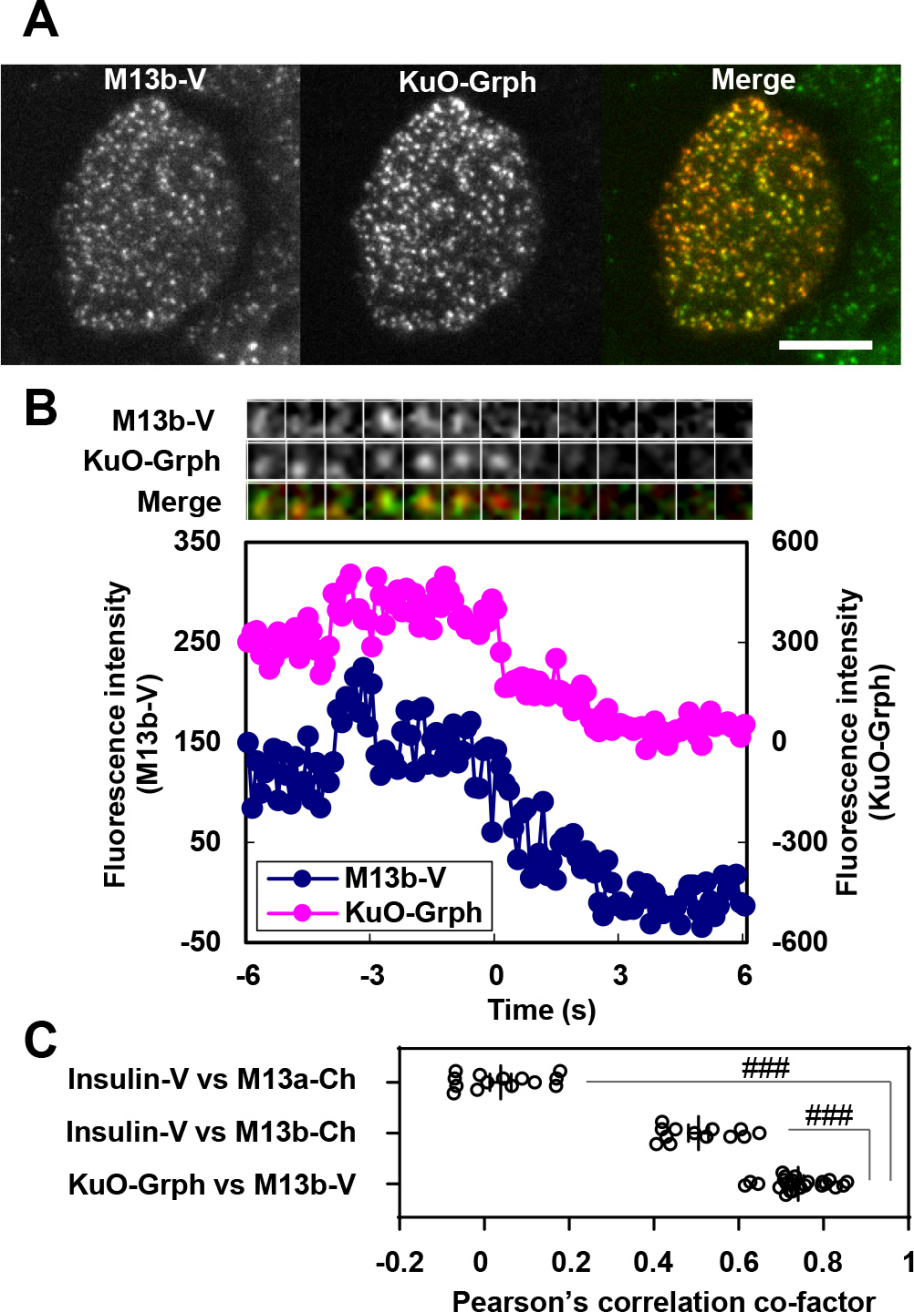
Munc13b accumulates on granuphilin-positive granules beneath the plasma membrane prior to fusion A: Granuphilin-null beta cells were infected with adenoviruses expressing Munc13b-Venus (M13b-V) and KuO-Granuphilin (Grph), and were observed under TIRF microscopy. Bar, 10 μm. B: The cells were stimulated by 60 mM KCl for 5 min under TIRF microscopy. A representative fluorescence intensity profile of Munc13b-V (blue) is shown for the case where the fluorescence of KuO-Grph drops upon granule fusion (red). A kymograph is shown at the top. C: Pearson’s correlation co-factors between Munc13a-Cherry (M13a-Ch) and Insulin-V, M13b-Cherry and Insulin-V, and M13b-V and KuO-Grph were calculated from TIRFM images, as shown in Supplementary Fig. 6A, Fig. 3A, and Fig. 6A, respectively (*n*=13~24 cells from 3~6 experiments). The TIRF 30×30 pixels images from cell adhesion regions after depolarization stimulation in each cell were used in the calculations. Note that the degree of colocalization between M13b-V and KuO-Grph is significantly higher than that between M13b-Ch and Insulin-V and that between M13a-Ch and Insulin-V. ^###^*P*<0.001 by one-way ANOVA followed by post hoc Tukey’s multiple comparisons test.

We then expressed Munc13b-Venus instead of Insulin-Venus in this granuphilin-null beta-cell line to monitor the dynamics of Munc13b and granuphilin simultaneously. Under TIRF microcopy, Munc13b-Venus was well colocalized with KuO-Granuphilin (Fig. 6A). Although granule fusion cannot be directly monitored in these cells, the resident type exocytosis from granuphilin-positive granules can be indirectly detected by the decrease in KuO-Granuphilin fluorescence upon fusion, as shown in Fig. 5B. Munc13b-Venus accumulated on most (88%, 22/25 events) of the granuphilin-positive granules showing a decrease in granuphilin fluorescence (Fig. 6B, Supplementary Video 3). The lag time between the peak of Munc13b-Cherry and the decline of KuO-Granuphilin was 2.20±0.37 s, similar to the lag time between the peak of Munc13b-Cherry and the decline of Insulin-Venus upon fusion (Fig. 3C). These findings suggest that Munc13b promotes exocytosis via its accumulation on granuphilin-positive docked granules. Consistent with this notion, the colocalization of Munc13b with granuphilin-positive granules is much higher than that with whole insulin granules (Fig. 6C), although we could not detect biochemical interaction between Munc13b and granuphilin in cells (Supplementary Fig. 7).

## Discussion

In the present study, we first show that among the Munc13 family proteins, Munc13a and the ubiquitous form of Munc13b are expressed in pancreatic beta cells, and that Munc13b primarily functions in insulin granule exocytosis. Although the role of Munc13b has never been investigated in pancreatic beta cells, our finding is consistent with the previous report showing that Munc13b plays a major role in granule exocytosis in chromaffin cells (Man *et al.*, 2015). However, it has been shown that even in Munc13a and Munc13b doubly deficient hippocampal neurons, dense-core granule release is not abrogated (van de Bospoort *et al.*, 2012). These findings suggest that a single isoform of Munc13 may function in a specific subtype of granule exocytosis. Indeed, we show that Munc13b downregulation specifically decreases the exocytosis of granules beneath the plasma membrane. Although both Munc13a and Munc13b are mainly located in the cytosol, only Munc13b is partly distributed and is stimulus-dependently accumulated on those granules just prior to fusion. On the other hand, Munc13a is stimulus-dependently translocated to the plasma membrane. It has been shown that Munc13a haplodeficiency primarily affects the exocytosis of granules stimulus-dependently recruited from the cell interior in mouse islet beta cells (Xie *et al.*, 2012). We also observed a tendency of the decrease in this passenger type exocytosis in Munc13a-KD MIN6 cells. It seems that Munc13b opens syntaxins that are already associated with granules, whereas Munc13a opens syntaxins that are free of granules on the plasma membrane. Consistent with this hypothesis, we recently found that the passenger type is mediated by another Rab27 effector, melanophilin, that specifically interacts with the open form of syntaxin-4 (Wang *et al.*, 2020). If so, different isoforms of Munc13 family proteins differentially act on granules displaying distinct prefusion behaviors.

However, Munc13b accumulates on only 40% of granules showing the resident type exocytosis. We previously showed by TIRF microscopy that among the granules showing the resident type exocytosis, half of them is granuphilin-positive and immobile before stimulation, whereas the other half of them is granuphilin-negative and more mobile, in MIN6 cells (Mizuno *et al.*, 2016). Therefore, we speculated that granule fusion with Munc13b accumulation corresponds to granuphilin-mediated, docked granule exocytosis. Indeed, we found that both the degree of colocalization and the ratio of accumulation prior to fusion between Munc13b-Venus and KuO-Granuphilin are much higher than those between Munc13b-Cherry and Insulin-Venus. The amount of Munc13b after stimulation may play a critical role in the exocytosis of those docked granules, since granuphilin imposes a fusion constraint by interacting with a fusion-incompetent, closed form of syntaxins-1~3 (Fukuda *et al.*, 2005; Torii *et al.*, 2002; Wang *et al.*, 2011). We previously showed that granuphilin forms a 180-nm cluster at the site of each docked granule (Mizuno *et al.*, 2016). Multiple granuphilin molecules are thought to be required for tethering insulin granules having a 350~400-nm diameter to the plasma membrane, although the exact molecular number is unknown. Thus, it is likely that a substantial number of Munc13b molecules are also required for priming of those docked granules. Although the accumulation of priming factors after stimulation will not determine the timing of synaptic transmission that occurs within less than 1 ms after the entry of Ca^2+^, it may determine that of granule exocytosis, which occurs much more slowly. In fact, it has been shown that SNARE proteins are assembled only shortly prior to insulin exocytosis in pancreatic beta cells, while they are preassembled before stimulation in the active zone of resting presynaptic boutons (Takahashi *et al.*, 2015). However, there is a several seconds-long lag time between Munc13b accumulation and fusion. It is possible that additional priming factors, such as the calcium-dependent activator protein for secretion (CAPS), are required for fusion, because it has been suggested that CAPS functions downstream of Munc13s in chromaffin granule exocytosis (Liu *et al.*, 2010).

Despite differences in function between Munc13a and the ubiquitous form of Munc13b, they share the domain structure consisting of one C1 domain, three C2 domains, and two Munc13 homology domains (MHDs), and they both have the highly conserved primary sequence of each domain (85–95%), except the N-terminal flanking sequence (~50%) and the C-terminal region containing the MHD2 and C2C domains (70–75%). The latter C-terminal region is thought to be responsible for catalyzing the conformational change of syntaxins, because it is sufficient to rescue secretory defects due to Munc13 deficiency in hippocampal neurons (Basu *et al.*, 2005) and to increase chromaffin granule secretion (Stevens *et al.*, 2005). This region might discriminate syntaxin isoforms that differentially mediate the different types of exocytosis. Alternatively, the most variable N-terminal region containing the C1, C2A, and C2B domains that have affinities to membrane phospholipids and Ca^2+^ may be involved in the stimulus-dependent translocation of Munc13 isoforms to the specific membrane compartments. Future research will seek to identify the molecular mechanism accounting for the differential function of Munc13 family proteins.

## Abbreviations

CAPS: calcium-dependent activator protein for secretion
GSIS: glucose-induced insulin secretion
GFP: green fluorescent protein
KD: knockdown
KI: knockin
KO: knockout
KuO: Kusabira Orange-1
MHD: Munc13 homology domain
RFP: red fluorescent protein
TIRF: total internal reflection fluorescence
