# Supplementary material for: Munc13b stimulus-dependently accumulates on granuphilin-mediated, docked granules prior to fusion
Source: Cell Struct Funct. 2022 Apr 6;47(1):31–41. doi: 10.1247/csf.22005 (PMC10511056; doi:10.1247/csf.22005)
Supplement: Supplementary file 1 — Supplementary Fig. 1 [file csf_47_22005_1.pdf]

Supplementary Figures

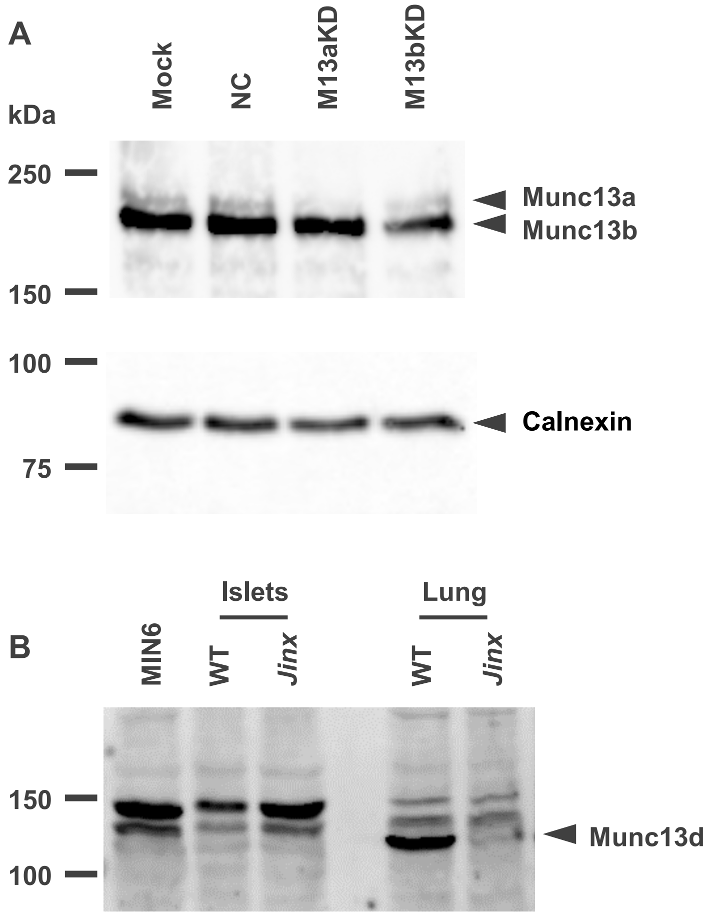

Supplementary Figure 1. Munc13a and Munc13b, but not Munc13c or Munc13d, are expressed in pancreatic islets

A: Mouse pancreatic islet cells were treated without (Mock) or with nonspecific control siRNA (NC) or siRNA against Munc13a (M13aKD) or Munc13b (M13bKD), as described in Fig. 1A. The protein extracts (20 μg) were electrophoresed for immunoblotting with anti-panMunc13 and anti-calnexin antibodies. B: The protein extracts (15 μg) from MIN6 cells, pancreatic islets, and lung from wild-type and Munc13d-deficient *Jinx* mice were electrophoresed for immunoblotting with anti-Munc13d antibody.
