# Supplementary material for: Munc13b stimulus-dependently accumulates on granuphilin-mediated, docked granules prior to fusion
Source: Cell Struct Funct. 2022 Apr 6;47(1):31–41. doi: 10.1247/csf.22005 (PMC10511056; doi:10.1247/csf.22005)
Supplement: Supplementary file 2 — Supplementary Fig. 2 [file csf_47_22005_2.pdf]

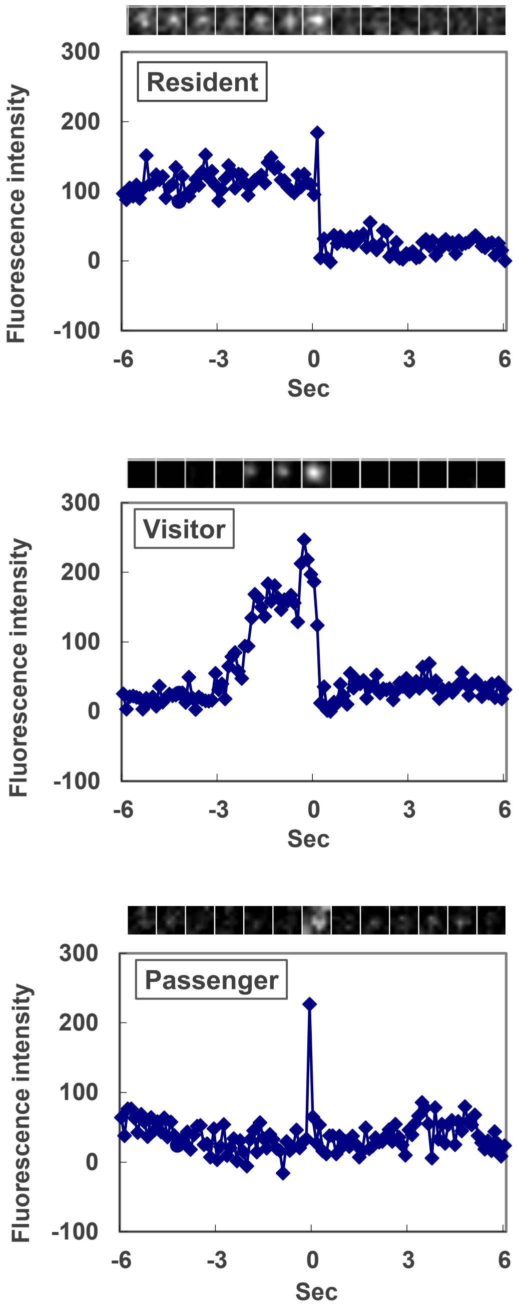

Supplementary Figure 2. Classification of insulin granule exocytosis observed by TIRF microscopy

Examples of fluorescence intensity profiles of resident, visitor, and passenger type exocytosis in cells expressing Insulin-Venus are shown with image sequences. A kymograph is shown at the top. The size of each frame is 6 × 6 pixels (1 pixel equals 0.16 μm) and the time interval is 1 s.
