# Supplementary material for: Munc13b stimulus-dependently accumulates on granuphilin-mediated, docked granules prior to fusion
Source: Cell Struct Funct. 2022 Apr 6;47(1):31–41. doi: 10.1247/csf.22005 (PMC10511056; doi:10.1247/csf.22005)
Supplement: Supplementary file 3 — Supplementary Fig. 3 [file csf_47_22005_3.pdf]

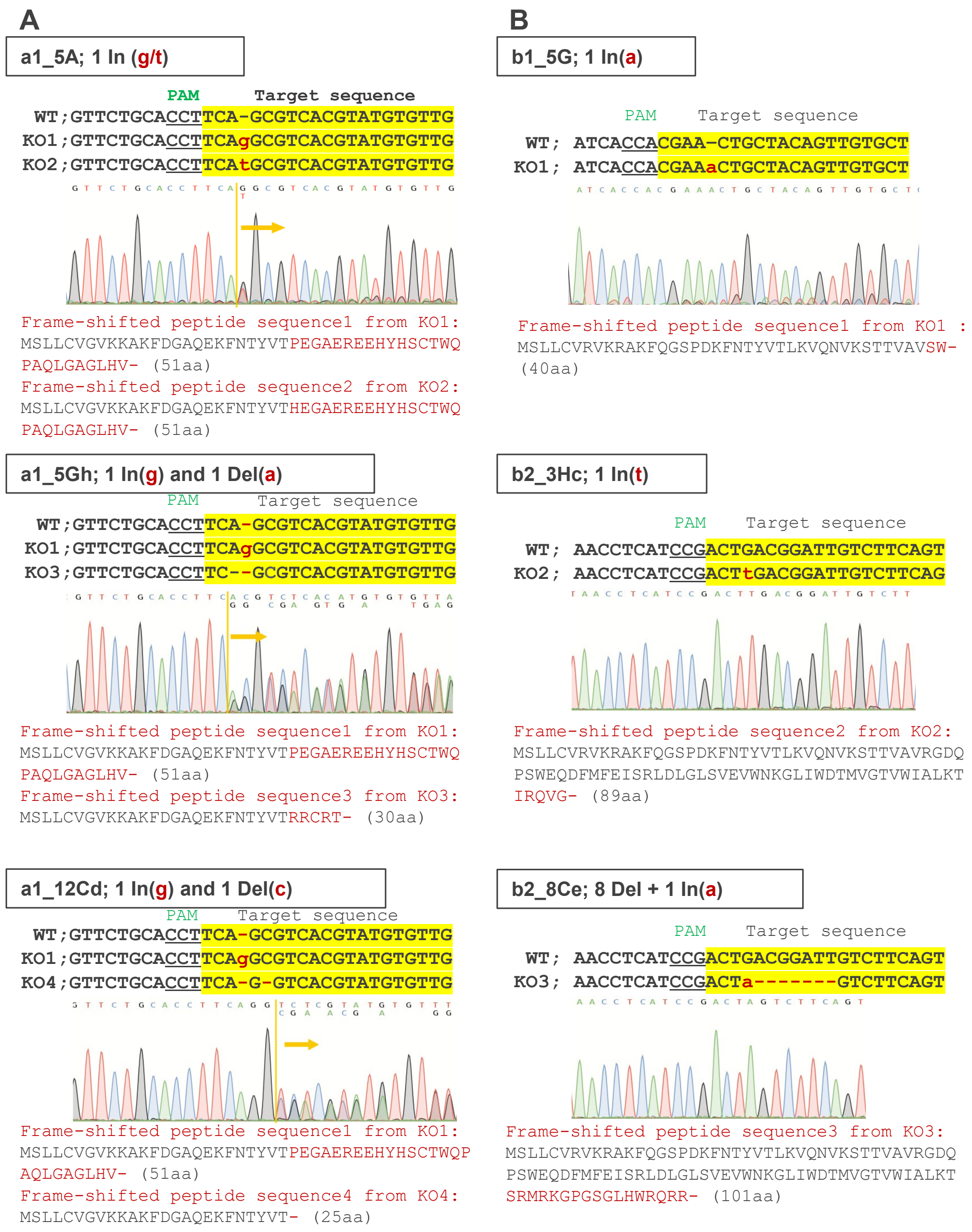

Supplementary Figure 3. Generation of Munc13a-null and Munc13b-null beta cell lines by CRISPR-Cas9 methods

Munc13a (A) or Munc13b (B) knockout (KO) MIN6 cells were generated using the CRISPR/Cas9 system. Shown are DNA sequences of genomic PCR products around the gRNA target sequences (yellow) from the cell clones shown in Fig. 2A: wild-type (WT) and identified indel mutations (red, top in each panel). Predicted amino acid sequences caused by the frameshift mutation up to the stop codon are also shown (red, bottom in each panel).
