# Supplementary material for: Munc13b stimulus-dependently accumulates on granuphilin-mediated, docked granules prior to fusion
Source: Cell Struct Funct. 2022 Apr 6;47(1):31–41. doi: 10.1247/csf.22005 (PMC10511056; doi:10.1247/csf.22005)
Supplement: Supplementary file 4 — Supplementary Fig. 4 [file csf_47_22005_4.pdf]

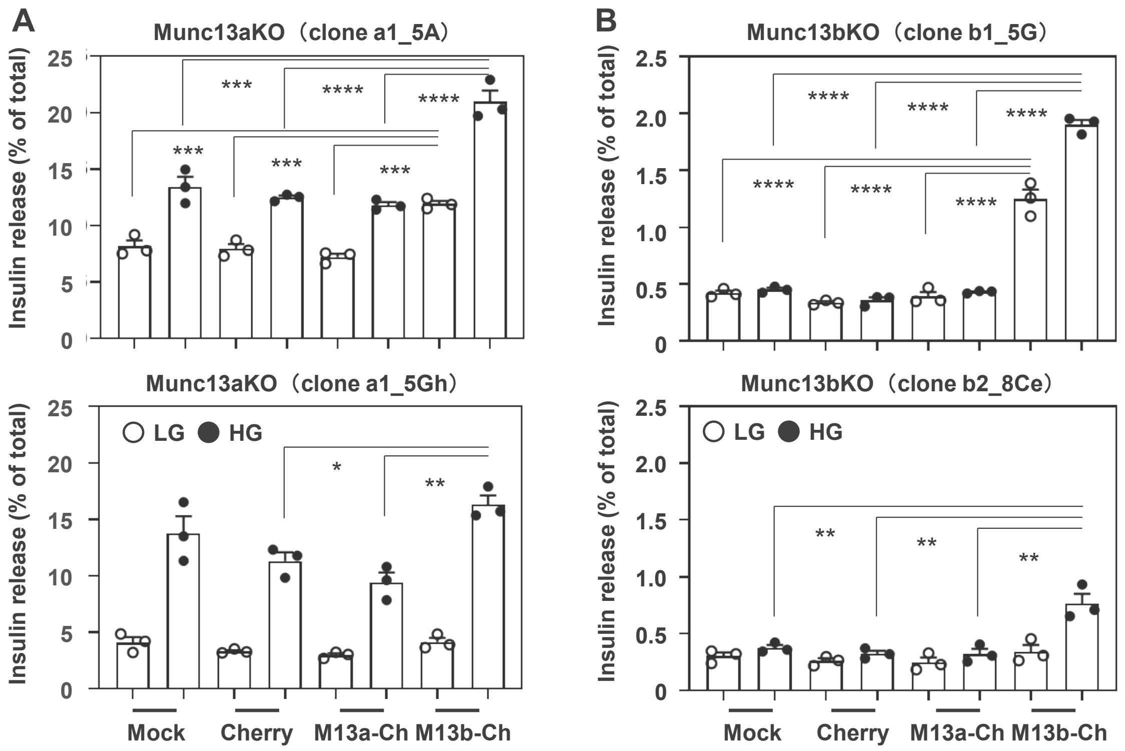

Supplementary Figure 4. Absence of Munc13b eliminates GSIS

MIN6 cell lines lacking Munc13a (clones a1_5A and a1_5Gh; Munc13aKO cells) or Munc13b (clones b1_5G and b2_8Ce; Munc13bKO cells) were infected by adenoviruses expressing Cherry, Munc13a-Cherry (M13a-Ch), or Munc13b-Cherry (M13b-Ch) under the conditions described in Supplementary Fig. 5. The cells were subjected to insulin secretion assays as described in Fig. 2B ($n = 3$). Note that expression of M13b-Ch, but not of M13a-Ch, augments (upper) or recovers (lower) GSIS in Munc13aKO and Munc13bKO cells, respectively. * $P < 0.05$, ** $P < 0.01$, *** $P < 0.001$, **** $P < 0.0001$ by one-way ANOVA followed by post hoc Tukey's multiple comparisons test.
