# Supplementary material for: Munc13b stimulus-dependently accumulates on granuphilin-mediated, docked granules prior to fusion
Source: Cell Struct Funct. 2022 Apr 6;47(1):31–41. doi: 10.1247/csf.22005 (PMC10511056; doi:10.1247/csf.22005)
Supplement: Supplementary file 5 — Supplementary Fig. 5 [file csf_47_22005_5.pdf]

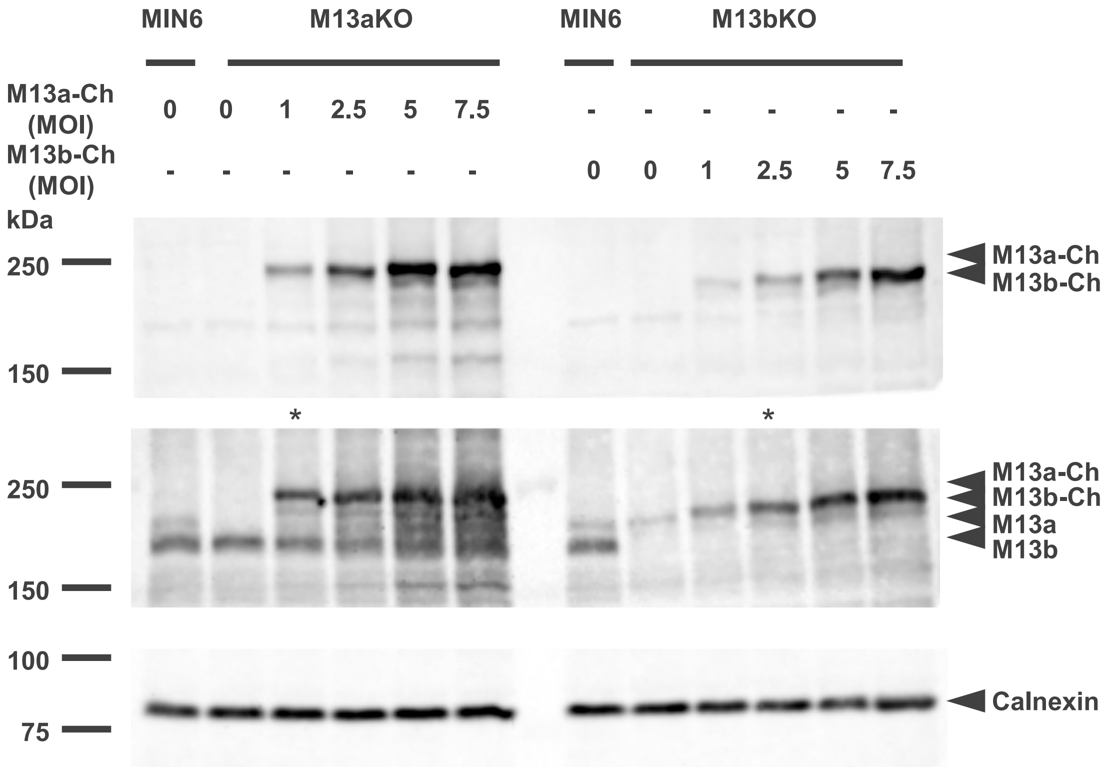

Supplementary Figure 5. MIN6 cells express approximately 2~3-fold higher levels of Munc13b compared with those of Munc13a

Munc13aKO and Munc13bKO cells were infected with different titers of adenoviruses expressing Munc13a-Cherry (M13a-Ch) and Munc13b-Cherry (M13b-Ch), respectively. The protein extracts (50 μg) from those cells and parental MIN6 cells were electrophoresed for immunoblotting with anti-RFP (upper), anti-panMunc13 (middle), and anti-calnexin (lower) antibodies. Note that the expression level of endogenous Munc13b is similar to that of M13b-Ch expressed by MOI 2.5 (right asterisk), and that this expression level is similar to that of M13a-Ch expressed by MOI 1 (left asterisk), which is 2~3-fold higher than that of endogenous Munc13a.
