# Supplementary material for: Munc13b stimulus-dependently accumulates on granuphilin-mediated, docked granules prior to fusion
Source: Cell Struct Funct. 2022 Apr 6;47(1):31–41. doi: 10.1247/csf.22005 (PMC10511056; doi:10.1247/csf.22005)
Supplement: Supplementary file 6 — Supplementary Fig. 6 [file csf_47_22005_6.pdf]

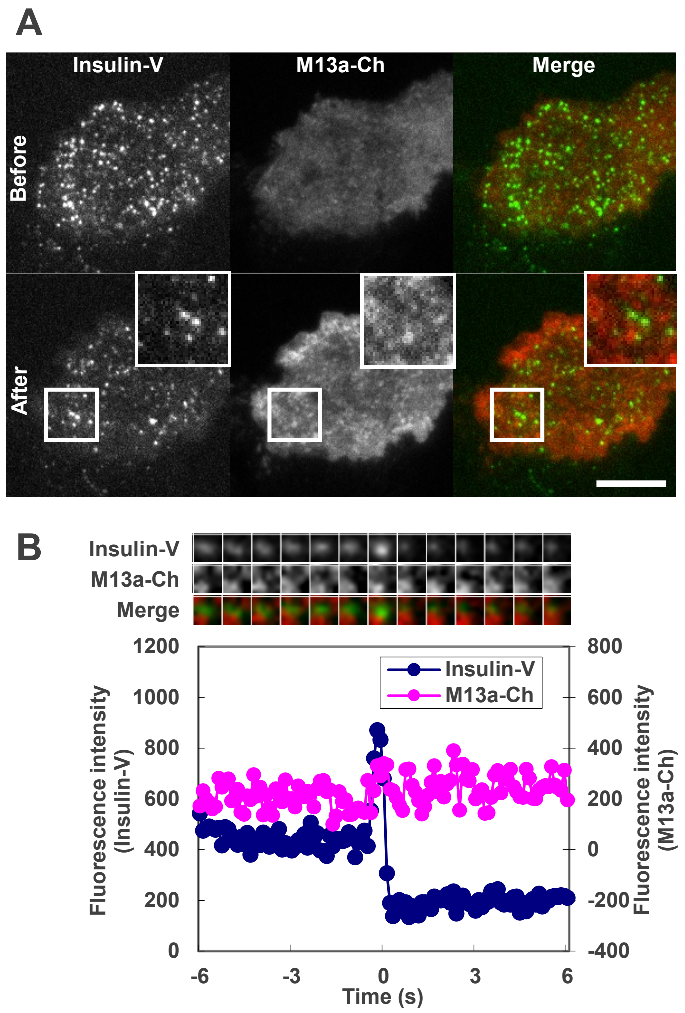

Supplementary Figure 6. Munc13a does not accumulate on granules beneath the plasma membrane but is translocated to the plasma membrane in a stimulus-dependent manner

A: Intracellular distributions of Insulin-Venus (V) and Munc13a-Cherry (M13a-Ch) expressed in Munc13aKO cells before and after 60 mM KCl stimulation were observed under TIRF microscopy. Insets represent 2×-magnification photomicrographs of a cell within the region outlined. Note that Munc13a is translocated to the plasma membrane in a stimulus-dependent manner, but is not colocalized with Insulin-Venus. Bar, 10 µm. B: Representative fluorescence intensity profiles of Insulin-V (blue) and M13a-Ch (red) are shown just before and after resident type exocytosis. A kymograph is also shown at the top.
