# Supplementary material for: Munc13b stimulus-dependently accumulates on granuphilin-mediated, docked granules prior to fusion
Source: Cell Struct Funct. 2022 Apr 6;47(1):31–41. doi: 10.1247/csf.22005 (PMC10511056; doi:10.1247/csf.22005)
Supplement: Supplementary file 7 — Supplementary Fig. 7 [file csf_47_22005_7.pdf]

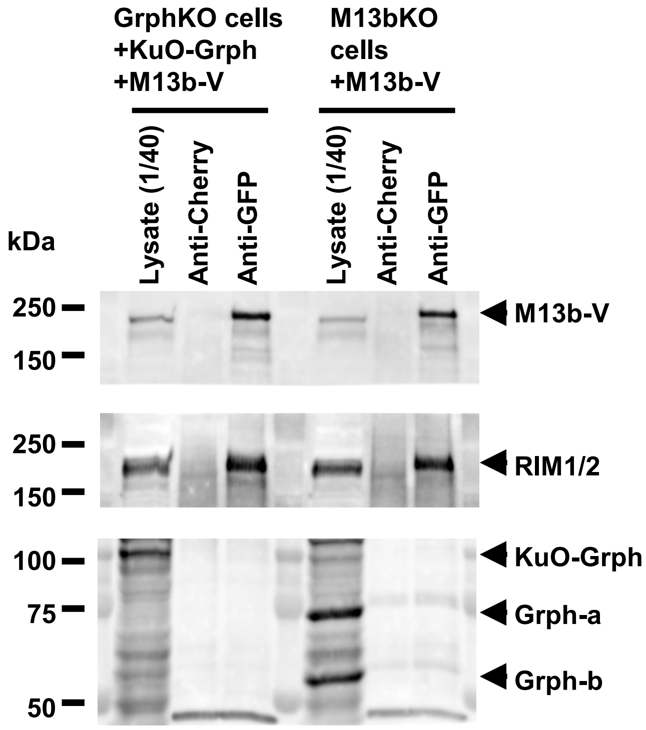

Supplementary Figure 7. Munc13b and granuphilin do not interact in cells

GranuphilinKO cells expressing KuO-Granuphilin (Grph) and Munc13b-Venus (M13b-V) and Munc13bKO cells expressing M13b-V by adenovirus (Ad) were immunoprecipitated with anti-Cherry or anti-GFP nanobody. The immunoprecipitates as well as 1/40 volume of the cell lysates were electrophoresed in 7% polyacrylamide gels, and were analyzed by immunoblotting with anti-GFP, anti-RIM1/2, and anti-granuphilin antibodies. Munc13b interacts with RIM1/2, but not with exogenous or endogenous granuphilin.
